# 

**Published:** 1916-09-09

**Authors:** 


### CONTENTS.

PAGE

**The Hospitals' Harvest Time** ... ... ... ... 533

**Sir Peter Eade** ... ... ... ... ... ... 534

**Hospital and Institutional News** ... ... ... 535

The Late Major Pell—The Responsibility of the Critic—The Conditions of Supply of Salvarsan—The Effective Demand for Annual Reports—Loyalty at Letterkenny—The Irish Local Government Board's Firm Policy—Workmen's Subscriptions in Wales—An Objection to Women Chauffeurs—The Exemption of a Medical Student—A New Mortuary Chapel—The War and Poor-Law Centralisation—An Addition to the Ward-Locker—A Practised Designer of Hospital Tableaux—The Holding-up of Sanatoria Buildings—Hospital Benefits by Inquest Fees—The 4th Southern Hospital's *Gazette* at Plymouth—A *Gazette's* First Birthday—Nurses' Recreation at Hull—This Week's Drug Market.

**Infantile Paralysis or Acute Anterior Poliomyelitis** ... ... ... ... ... ... 539

Its Symptoms and Treatment.

**King Edward VII. Welsh National Memorial Association** ... ... ... ... ... ... 540

The Fourth Annual Report.

**Human Temperaments** ... ... ... ... 541

X.—The Jealous Temperament.

**Our Hospitals and the National Relief Fund** 543

More Facts and Figures.

PAGE

**Experimental Tuberculous Meningitis** ... ... 542

**The Matrons' and Sisters' Department** ... 545

The Future of British Nursing.

Round the Hospitals.

A Notable Matron's Retirement—Honours for Nurses—An Interesting Address—Complaints of Familiarity—The Patient's Point of View.

**The National Poor-Law Officers' Association** 547

Why it Cannot Represent Trained Nurses.

**The Editor's Letter-Box** ... ... ... ... 549

Some Hospital Statistics: Their Use and Value—Anthrax in the Shaving-brush—Japanese Shave without Soap.

**The London Hospital** ... ... ... ... ... ... 549

Work of the Past Quarter.

**Smaller General Hospitals** ... ... ... ... 550

The Horton Infirmary, Banbury.

**The Book Market** ... ... ... ... ... ... 550

**Passing Events and Topics** ... ... ... ... vi

**Matrons' and Sisters' Appointments** ... ... vi

**Gifts to Hospitals** ... ... ... ... ... ... vi

**The Institutional Worker** ... ... (Suppl.) 1

The Keeping of Medical and Surgical Records: Answer to the August Question. Question for September.

